# Tunable Schottky Barrier and Interfacial Electronic Properties in Graphene/ZnSe Heterostructures

**DOI:** 10.3389/fchem.2021.744977

**Published:** 2021-10-01

**Authors:** Wenjun Xiao, Tianyun Liu, Yuefei Zhang, Zhen Zhong, Xinwei Zhang, Zijiang Luo, Bing Lv, Xun Zhou, Zhaocai Zhang, Xuefei Liu

**Affiliations:** ^1^ College of Physics and Electronic Science, Guizhou Normal University, Guiyang, China; ^2^ Key Laboratory of Low Dimensional Condensed Matter Physics of Higher Educational Institution of Guizhou Province, Guizhou Normal University, Guiyang, China; ^3^ Beijing Institute of Space Science and Technology Information, Beijing, China; ^4^ College of Information, Guizhou University of Finance and Economics, Guiyang, China

**Keywords:** 2D heterojunction, Schottky barrier height, horizontal and vertical strain, bader charge, density function theory

## Abstract

With a direct bandgap, two-dimensional (2D) ZnSe is a promising semiconductor material in photoelectric device fields. In this work, based on first-principles methods, we theoretically studied the modulation of the Schottky barrier height (SBH) by applying horizontal and vertical strains on graphene/ZnSe heterojunction. The results show that the inherent electronic properties of graphene and ZnSe monolayers are both well-conserved because of the weak van der Waals (vdW) forces between two sublayers. Under horizontal strain condition, the n(p)-type SBH decreases from 0.56 (1.62) eV to 0.21 (0.78) eV. By changing the interlayer distance in the range of 2.8 Å to 4.4 Å, the n(p)-type SBH decreases (increases) from 0.88 (0.98) eV to 0.21 (1.76) eV. These findings prove the SBH of the heterojunction to be tuned effectively, which is of great significance to optoelectronic devices, especially in graphene/ZnSe-based nano-electronic and optoelectronic devices.

## Introduction

Ever since graphene was exfoliated experimentally in 2004 Novoselov et al. (2004), owing to its unique advantages, such as high charge mobility at room temperature and excellent Hall effect Novoselov et al. (2005), Zhang et al. (2005), it has attracted many theoretical and experimental attentions (Olabi et al., 2021; cao et al., 2018; Wang et al., 2020; Niu et al., 2020). Nevertheless, ZnSe as a zero bandgap material was not well-developed in graphene photonics and optoelectronics (Bonaccorso et al., 2010). In this regard, a lot of efforts have been devoted to exploring other novel two-dimensional (2D) crystal structures, and some new 2D materials were prepared (Naguib et al., 2013; Niu and Li, 2015; Liao et al., 2020). Among them, ZnSe is a direct bandgap 2D semiconductor material with a wide gap of 3.24 eV by using Heyd–Scuseria–Ernzerhof (HSE) functional (Krukau et al., 2006), and it has attracted more and more attention in recent years. For instance, ZnSe was proved to be a potential material as inorganic scintillators Jagtap et al. (2019), as well as a cathode material of battery (Zhu et al., 2019). Besides, 2D ZnSe crystals have exhibited other interesting properties, including piezoelectric and dopant-induced semi-metallic tunable bandgap Liu et al. (2014), Yu et al. (2019), Khan et al. (2020), Sun et al. (2020), enabling its great potential applications in nano-electronics and optoelectronics fields. Furthermore, the energy bands of ZnSe meet the conditions of photocatalytic water splitting. Hence, it is also expected to be developed in new energy devices (Rubini et al., 2001; Hazrati et al., 2008; Yao et al., 2020).

The van der Waals (vdW) heterostructure, as proposed by Geim and Grigorieva (2013), is composed of multiple 2D atomic layers without chemical bonds between them. Due to the weak interlayer vdW forces, heterojunction often retains the individual electronic and optical properties of a single layer, and some new physical properties may be obtained at the interface. Therefore, in recent years, the 2D vdW heterojunction has attracted extensive attention in the field of electronic and optoelectronic devices (Zhang et al., 2020a; Guo et al., 2020; Zhu et al., 2021). Many scholars have studied vdW heterojunction based on ZnSe, such as AlP/ZnSe, ZnSe_2_/ZnSe, and CdS/ZnSe heterostructures (Dinger et al., 1999; Xiong and Zhou, 2019; Liu et al., 2020a). However, all the interfaces were constructed by two semiconductors, rather than metal/semiconductor heterojunction. In fact, the interface properties of the semiconductor/metal heterojunction such as the charge transfer and Schottky barrier play a vital role in the device performance (Zhang et al., 2020b; Robertson et al., 2020; Chen et al., 2021). How is the charge transfer between a 2D ZnSe nanosheet and graphene? How to tune the charge transfer and other electronic properties of the graphene/ZnSe interface? These questions have not been understood well yet. Hence, it is very essential to understand the interfacial properties of the ZnSe-based metal–semiconductor heterojunctions.

Besides, the 2D heterojunction Schottky barrier height (SBH) can be controlled through interlayer coupling, electric field, biaxial strain engineering, and atomic doping (Si et al., 2016; Zhang et al., 2018; Zhou et al., 2018; Zhang et al., 2020a; Nguyen et al., 2020; Liu et al., 2021). The modulation of SBH would improve the carrier transmission rate and change the type of Schottky contact. Although many studies on graphene-based vdW heterojunction have been published Georgiou et al. (2012), Si et al. (2016), Qiu et al. (2020), the electronic properties of the graphene/ZnSe heterostructure have not been clearly understood yet, as well as the SBH tunability under horizontal and vertical strain. In this study, we constructed a graphene/ZnSe heterojunction and calculated the electronic properties systematically. Our findings indicated the SBH of graphene/ZnSe could be effectively tuned by applying horizontal strain and vertical strain, which has potential applications in nano-mechanics, transistors, piezoelectric, and optoelectronics applications.

## Computational Methods

In this study, all the calculations are based on the density functional theory (DFT) and projector augmented wave (PAW) Blöchl (1994), as carried out in the Vienna ab initio simulation package (VASP) (Kresse and Furthmüller, 1996). The Perdew–Burke–Ernzerhof (PBE) method based on the generalized gradient approximation (GGA) was used to describe exchange–correlation potential (Perdew et al., 1996). The geometric optimization and electronic property calculations were accomplished by applying an energy cutoff of 520 eV. The total energy convergence was set at 10^–5^ eV. The atomic structures considered were fully optimized until the convergence of force of 0.01 eV/Å. The Γ-centered Monkhorst–Pack Monkhorst and Pack (1976) is used to sample the reciprocal space with a grid density of 5 × 5×1. The weak interaction was corrected between sublayers by using Grimme’s DFT-D3 method (Grimme, 2006). We used a vacuum thickness of 20 Å to avoid bonding between the periodic sublayers (Liu et al., 2020b). Dipole correction was considered to reduce the error due to the asymmetry of the graphene/ZnSe interface along the *Z*-direction. The VASPKIT was used to do part of data post-processing (Wang et al., 2021).

The binding energy is one of the physical quantities that determine the heterojunction structural stability (Gélinas et al., 2011). Therefore, to verify the stability, we calculated the binding energies for graphene/ZnSe vdW heterostructure as follows (Zhao-Fu et al., 2014; Guo et al., 2020) (1):

$$E_{b}=\frac{E_{graphene/ZnSe}-E_{graphene}-E_{ZnSe\;}}{A}.$$ (1)

Here, $E_{b}$ is the heterojunction binding energy; $E_{graphene/ZnSe}$ represents the total energy of the heterostructure; $E_{graphene}$ and $E_{ZnSe}$ are the total energy of the graphene and ZnSe monolayer, separately; and *A* is the interface area in the x-y plane.

## Results and Analysis

### Structural Properties

**FIGURE 1:**
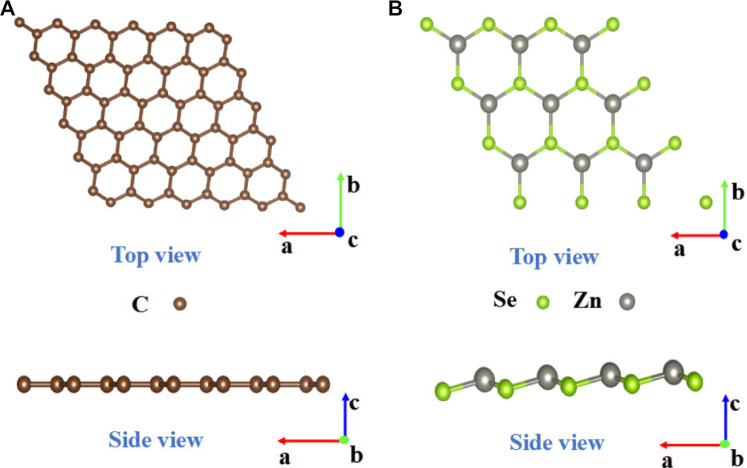
**(A)** Top and side views of a 5 × 5 graphene, and **(B)** the top and side views of a 3 × 3 ZnSe supercell. The brown spheres represent C, the gray spheres represent Zn, and the green spheres represent Se.

**FIGURE 2:**
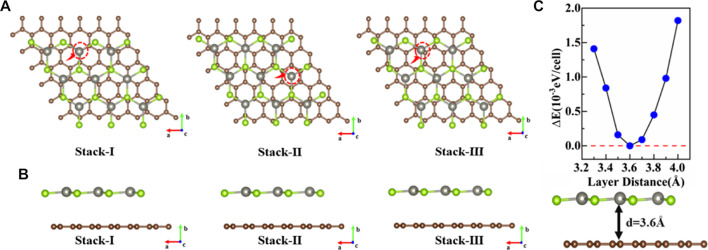
**(A)**, **(B)** Three stacked patterns of top and side views. **(C)** Relationship between layer spacing and binding energy of graphene/ZnSe.

Before constructing the graphene/ZnSe heterojunction, the lattice constants of graphene and ZnSe are relaxed to be 2.46 Å and 4.07 Å, respectively, being in great agreement with the data in the literature (Priyadharsini et al., 2016; Yang et al., 2018). To reduce the mismatch, the graphene/ZnSe heterostructure was constructed by using a 5 × 5 supercell of graphene and a 3 × 3 supercell of ZnSe, as shown in Figure 1. It is noted that the lattice constants in the heterostructure for graphene and ZnSe are 12.3 Å and 12.21 Å, leading to a lattice mismatch of only 0.73%. To obtain the most stable heterojunction, we considered three stacking patterns based on the main high symmetry nature, as shown in Figure 2A. The binding energy of three stacking patterns is shown in Table 1. The stack-III pattern with a binding energy of −2.01 meV ${\text{Å}}^{-2}$ is the most stable heterojunction. Thus, in the following calculation, we only considered the stack-III pattern.

**TABLE 1:** Binding energy of three stacking patterns.

Stack type	E_graphene_/_ZnSe_ (eV)	*E*_*graphene*_ (eV)	*E*_*ZnSe*_ (eV)	*E*_*b*_ (meV Å^−2^)
Stack-I	−514.61	−461.65	−49.41	−1.77
Stack-II	−514.70	−461.65	−49.41	−1.81
Stack-III	−515.11	−461.65	−49.41	−2.01

We further investigated the binding energy of stack-III pattern under different interlayer distances, as shown in Figure 2C. The results of relative energy indicated the most stable interlayer distance is 3.6 Å.

### Electrical Properties

**FIGURE 3:**
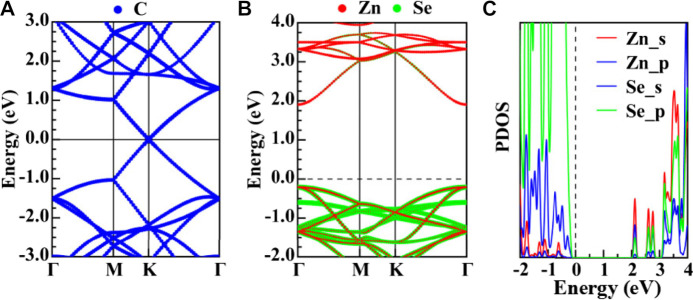
**(A)** Energy band diagram of graphene. **(B)**, **(C)** Projected energy band diagram and density of states of ZnSe, respectively.

As expected, graphene shows a metallic nature with the Dirac point located at the K point and crossed by the Fermi energy level, as shown in Figure 3. As for ZnSe, the valence band maximum (VBM) is at the Γ point, as well as the conduction band minimum (CBM), indicating that ZnSe is a direct bandgap semiconductor, with a bandgap value of 2.11 eV with PBE. According to the projected density of states (PDOS), the VBM of ZnSe was mainly contributed by the p-orbitals of Zn and Se, while the CBM was mainly contributed by the s-orbital electrons of Zn.

As well known, the PBE method would underestimate the bandgap of semiconductors, and the HSE method can solve this problem extremely well. However, both the PBE and HSE would predict the same band structure shape and variation trends of the ZnSe monolayer under different strains, with limited resources, so we use the PBE method to calculate all properties of the considered systems. Next, we further researched the electronic properties of the graphene/ZnSe heterojunction by figuring out the band structures and density of states under different horizontal or vertical strains (Phuc et al., 2017).

**FIGURE 4:**
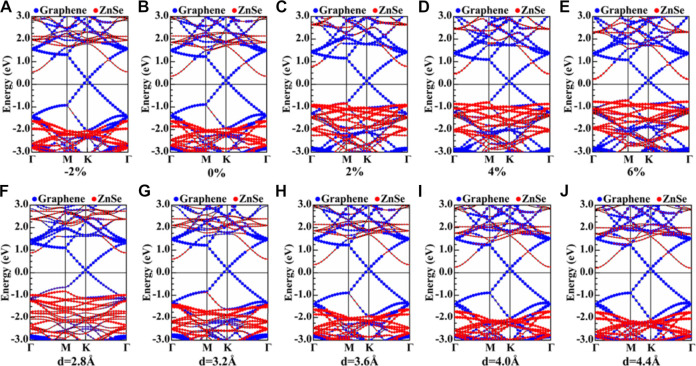
**(A–E)** Projected band structures of the graphene/ZnSe heterojunction under horizontal strain. **(F–J)** Projected band structures of the graphene/ZnSe heterojunction under vertical strain.

**FIGURE 5:**
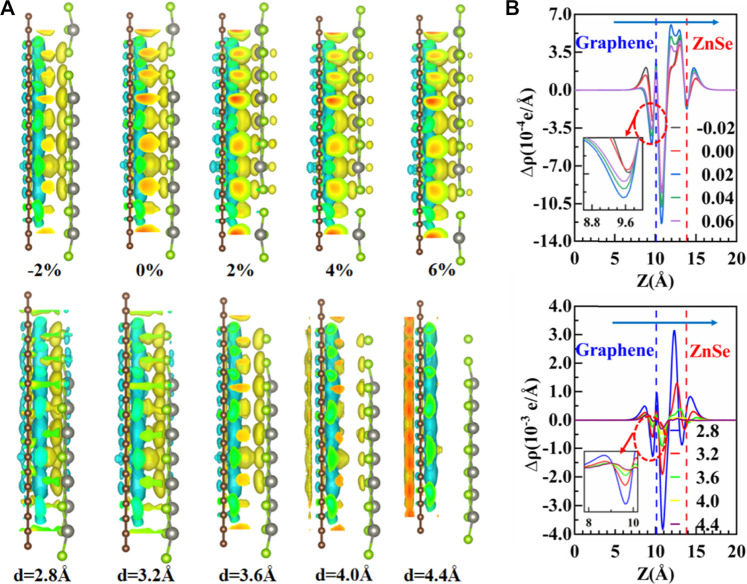
**(A)** On behalf of the charge density difference of the graphene/ZnSe system under different horizontal strains and vertical strain, yellow and cyan represent the accumulation and depletion of electrons, respectively. **(B)** PCDD under horizontal strain and vertical strain.

In this study, we applied horizontal strain from −2% to +6% on the graphene/ZnSe heterojunction, with a span of 2%. The positive value indicates tensile strain, while the negative value represents compressive strain. The reason why more negative strains are not considered is that a much larger strain is hard to be experimentally achieved, and it will also make the nanomaterials nonstable (Liao et al., 2020). When the compressive strain is less than −2%, the heterojunction optimization was failed. In general, tensile strain is relatively easier than that of compressive strain to implement in engineering. Hence, we only consider the −2% horizontal compression strain but a tensile strain of 6%. The projected band structures of the graphene/ZnSe heterojunction under different horizontal strains are shown in Figures 4A–E. From the figure, the Dirac point is well-maintained and the Fermi level is fixed at the K point. With the increase in the tensile strain from 0 to 6%, the bandgap decreases from 1.98 to 0.99 eV, and the bandgap always maintains a direct bandgap. The electron transfer ability of graphene to ZnSe is weakened, with the increased horizontal tensile strain smaller than 4%. When the horizontal tensile strain is larger than 4%, the electron transfer ability of graphene to ZnSe is enhanced. When horizontal or vertical compressive strain is applied, the CBM of ZnSe is moved up. Additionally, it seems that the horizontal compressive strain has a smaller effect than vertical strain on the VBMs, as shown in Figures 4A, F, G. Under tensile strain, both the VBMs and CBMs would shift up (down) for horizontal (vertical) cases, except for a horizontal strain of 6% (see Figure 4E). These different changes of CBM and VBM would lead to the bandgap change with the external strains. The results mean that both the vertical and horizontal strain plays a pivotal role in tuning the electronic properties of the graphene/ZnSe heterojunction. These phenomenons are resulting from the charge redistribution between the two sublayers under different strains (Liu et al., 2019; Liu et al., 2020b), as verified in Figure 5.

Next, to investigate the charge transfers and charge redistribution more clearly, the charge density difference of the graphene/ZnSe heterojunction was analyzed under different horizontal and vertical strains, as shown in Figure 5A. The charge density difference shows charge accumulation in the interface region near ZnSe and depletion in the interface region close to the graphene, which suggests a charge transfer from graphene to ZnSe. But, the variations in charge redistributions under different strain conditions are found, which leads to the different change regularity of band structures.

To see the charge redistribution, the plane-averaged charge density difference (PCDD) was calculated as follows (Zhang et al., 2015):

$$\Delta \rho =\rho_{graphene/ZnSe}-\rho_{graphene}-\rho_{ZnSe}.$$ (2)

**FIGURE 6:**
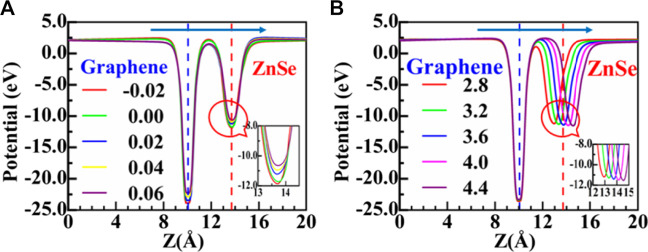
**(A**, **D)** Variation trend of CBM, VBM, and Fermi energy levels E_F_ with applied strain. **(B**, **E)** Variation of $\Phi_{n}$ , $\Phi_{p}$ , bandgap with the strain. **(C**, **F)** Amount of electron transfer from graphene to ZnSe as a function of the strain.

In this case, $\rho_{graphene/ZnSe}$ , $\rho_{graphene}$ , and $\rho_{ZnSe}$ are the PCDD of the graphene/ZnSe heterojunction, the isolated graphene single layer, and the isolated ZnSe, respectively. The PCDD curve is depicted in Figure 5B; when the horizontal strain is applied, charge transfer is tunable and agrees with the results of Bader charge analysis (Henkelman et al., 2006), as shown in Figure 6A, the charge is mainly concentrated on the surface near the ZnSe of layers. While the vertical strain is applied, as expected, the smaller the interlayer distance, the stronger coupling between ZnSe and graphene was found, leading to more electrons being transferred from graphene to ZnSe. The PCDD curve also proves the accuracy of the analysis of the band structure results.

**FIGURE 7:**
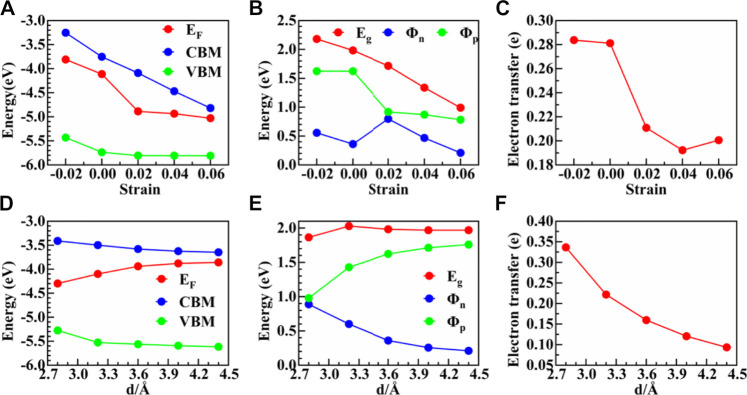
**(A)**, **(B)** Average electrostatic potential along the *z*-direction for the heterostructure under different horizontal strains and vertical strains, respectively.

In Figure 7C, we further depict the plane-averaged electrostatic potentials of the graphene/ZnSe interface under different horizontal and vertical strains. The graphene layer has a deeper potential than the ZnSe layer. The potential difference of the graphene/ZnSe heterostructure is 11.92 eV under an equilibrium distance of d = 3.6 Å, which further proves that the electrons are transferred from graphene to ZnSe. As shown in Figure 7A, horizontal strain ranges from −2% to +6%, the potential difference between graphene and ZnSe monolayers is tuned effectively, indicating that the ability of charge transfer can be modulated by applying horizontal strain. At the tensile strain of 2%, the potential difference reached 12.26 eV, indicating that the graphene/ZnSe vdW heterojunction should be able to maintain the high carrier mobility of graphene and promote the development of new high-performance nano-electronic devices. As shown in Figure 7B, under a vertical strain, it shows the potential difference between graphene and ZnSe under an interlayer distance of 2.8 Å and 4.4 Å is 12.38 and 11.78 eV, respectively. In other words, with the increase in the interlayer distance, the potential differences between graphene and ZnSe decrease, leading to the charge transfer from the graphene layer to the ZnSe layer being reduced, which is confirmed by the Bader charge analysis shown in Figure 6B.

### Tunability of SBH Under Strain

The SBH of the metal/semiconductor interfacial system is an important parameter (Zhang et al., 2020b; Robertson et al., 2020; Chen et al., 2021). Therefore, it is necessary to study the SBH of the graphene/ZnSe interface to better understand this heterojunction.

Based on Bardeeen’s (Bardeen, 1947) Schottky–Mott model of metal/semiconductor heterostructures, the n-type and p-type Schottky barriers are determined as follows:

$$\Phi_{n}=\;\mathrm{CBM}-E_{F}$$ (3)

$$\Phi_{p}=\;E_{F}-\mathrm{VBM}\text{,}$$ (4)

where the $\Phi_{n}$ denotes n-type SBH, $\Phi_{p}$ is p-type SBH, and E_F_ is the Fermi level. Without any horizontal or vertical strain, $\Phi_{n}$ and $\Phi_{p}$ of the graphene/ZnSe heterostructure are 0.36 and 1.62 eV, respectively, indicating that graphene/ZnSe vdW ( $\Phi_{n}$ < $\Phi_{p}$ ). As is well known, SBH and contact types of the heterojunction can be controlled by applying strain (Liu et al., 2019; Liu et al., 2020c). Therefore, we further investigate the effects of SBH and its tunability of the graphene/ZnSe heterojunction by applying the horizontal and vertical strains.

As shown in Figure 7A, the results indicate that CBM and VBM are decreased (increased) with the increase in horizontal tensile (compressive) strain and the Fermi energy level, but with a different change degree. Thus, the heterojunction bandgap was adjusted in a large range, indicating that the horizontal strain is an effective method of regulating the SBH. As is depicted more intuitively in Figure 7B, the results show both the n-type and p-type SBH was changed obviously under different horizontal strains. For instance, the n-type SBH can change from 0.56 to 0.21 eV, and the p-type SBH is decreased from 1.62 to 0.78 eV according to the applied strain values, but the n-type SBH contact is always maintained. Although the Ohmic contact is not realized, the large decrease in SBH would enhance the performance of the graphene/ZnSe-based nanodevices.

It should be noted that variation of heterojunction layer spacing can be achieved by experimental techniques (Zhang et al., 2014), which have been diffusely used to regulate the electronic properties and SBH of the vdW heterostructures. In this study, to understand how vertical strain regulates the graphene/ZnSe heterojunction SBH, the interlayer distance of the graphene/ZnSe heterojunction was changed from 2.8 Å to 4.4 Å. In Figures 7D, E, the VBM and CBM are decreased with the increase in the interlayer distance. In contrast, the Fermi energy level is shifted up until the interlayer is larger than 3.6 Å, leading to the SBH of n-type and p-type changing obviously, as shown in Figure 7E, and by changing the interlayer distance in the range of 2.8 Å to 4.4 Å, the n(p)-type SBH decreases (increases) from 0.88 (0.98) eV to 0.21 (1.76) eV. Thus, for the graphene/ZnSe heterostructure, the n-type Schottky is still maintained. Based on our results in Figure 7E, it can be informed that an n-type–to–p-type contact would be formed if the interlayer distance is smaller enough. Therefore, after the strain adjustment, graphene can be used as an ideal electrode material for ZnSe, and the SBH can be significantly tuned by applying both lateral and vertical strains, which is expected in the graphene/ZnSe-based Schottky devices.

## Conclusion

In conclusion, we have systematically studied the electronic properties and the efficient modulations of SBH of the vdW graphene/ZnSe heterostructure by DFT calculations. The band structures, the charge density differences, and the Bader charge transfer are studied in detail. The results show that the electrons will be transformed from graphene to ZnSe, and the transfer amount can be tuned effectively by applying both horizontal and vertical strains. As a consequence, the positions of CBM and VBM as well as Fermi energy level will be changed with the strain, and thus, the SBH is modulated effectively. These findings would provide useful guidance for designing controllable graphene/ZnSe-based Schottky nanodevices.

## Data Availability

The original contributions presented in the study are included in the article/supplementary material; further inquiries can be directed to the corresponding authors.
